# Urban Spontaneous Plants and Vegetation: Advantages and Management Challenges

**DOI:** 10.3390/plants15101576

**Published:** 2026-05-21

**Authors:** Francesca Bretzel, Daniela Romano

**Affiliations:** 1 Research Institute on Terrestrial Ecosystems, National Research Council, 56124 Pisa, Italy; francesca.bretzel@cnr.it; 2 NBFC (National Biodiversity Future Center), 90133 Palermo, Italy; 3 Department of Agriculture, Food and Environment (Di3A), University of Catania, 95131 Catania, Italy

**Keywords:** biodiversity, ecosystem services, habitats, plant traits, plant life forms, spontaneous colonisation, urbanisation

## Abstract

Urbanisation has led to dramatic alterations in pre-existing natural environments, resulting in several subsequent phenomena, such as the disappearance of habitats suitable for many plant and animal species and the concurrent arrival of generalist and non-native species, contributing to environmental homogenisation. Towns and cities serve as crossroads for transport, people, and animals, making them susceptible to colonisation by many types of plant species, dispersed either intentionally or unintentionally by these biotic vectors. Abiotic vectors, such as wind and water, also influence the composition of vegetation assemblages. Urban spontaneous vegetation occurs in (1) undisturbed areas, including brownfield sites, commons, and marginal lots, and (2) disturbed sites, such as green areas, parks, lawns (not subject to weeding), ancient monuments and walls, peripheral and industrial areas, and railways. When disturbance occurs, vegetation remains at early successional stages. Within this framework, with the aim of comparing existing contradictions and identifying knowledge gaps, we reviewed the literature on the characteristics of spontaneous plants and vegetation in urban areas, the different habitats in which they grow, the ecosystem services they provide, and management strategies, considering human perception. Our results highlight that studies on spontaneous plants are well-developed in terms of botany and ecology; however, some gaps remain, particularly regarding their integration into urban design and maintenance practices. Concerning public perception and acceptance, cultural and geographical differences emerged that deserve further investigation. In conclusion, spontaneous plants can represent a valuable heritage for cities, helping to address the challenges posed by the climate crisis.

## 1. Introduction

Urbanisation has led to profound modifications of pre-existing natural environments, triggering a series of interconnected processes, including the loss of habitats suitable for many plant and animal species and the concurrent spread of generalist and alien species, ultimately resulting in environmental homogenisation [1]. Towns and cities function as important crossroads for transport, people, and animals, which makes them particularly susceptible to colonisation by a wide variety of plant species, dispersed either intentionally or unintentionally by biotic vectors. Abiotic vectors, such as wind and water, also play a significant role in shaping the composition of urban vegetation assemblages. Spontaneous vegetation occurs in undisturbed or low-managed open urban areas, including brownfields, commons, marginal lots, lawns and turfs not subjected to weeding, ancient monuments and walls, peripheral industrial areas, and railway infrastructures. In such contexts, plant succession may develop over time, making it possible to estimate the age of spontaneous urban vegetation based on the plant species present [2]. By contrast, the dynamic nature of urban environments, often characterised by recurrent and intense disturbances, frequently interrupts successional processes, maintaining vegetation at early successional stages. These habitats are typically dominated by herbaceous swards and shrub communities, which provide important resources and shelter for a wide range of insect and mammal species [3].

Urbanisation often occurs at the expense of fertile alluvial soils that were formerly cultivated. These soils may retain seeds and buds of crops and their associated weeds, resulting in a rich soil seed bank that enables species with different regeneration strategies to establish following disturbance [4]. In some cases, cities can therefore act as reservoirs of biodiversity not present in the surrounding countryside, which may be degraded by intensive agricultural practices. Remnants of spontaneous vegetation, such as woodlands, wetlands, and grasslands, may persist within the urban matrix, incorporated into the expanding urban fabric. These relict habitats can hold significant conservation value, providing refuge for threatened and endangered organisms [5].

Through these processes, urban environments can host highly heterogeneous spontaneous floras. Assemblages of native and exotic species give rise to novel ecosystems that are absent from natural settings and are often referred to as “novel wilderness”. By contrast, “ancient wilderness” denotes long-lasting remnants of vegetation that have persisted relatively intact despite being enclosed within urban boundaries [6].

Urban flora is shaped by multiple interacting stressors, including: (i) habitat transformation, leading to habitat loss; (ii) fragmentation caused by grey infrastructure; (iii) urban environmental conditions, such as pollution, recurrent disturbance, and soil sealing; and (iv) human preferences and management practices [7]. Urban plants are generally thermophilic and tend to favour alkaline, nutrient-rich conditions, particularly environments characterised by high nitrogen availability. With respect functional types (FTs), therophytes—typically associated with high soil fertility and frequent disturbance—along with trees, are the most prevalent, whereas geophytes are comparatively underrepresented. In terms of functional traits, species producing heavier seeds and attaining greater plant height are generally favoured in urban environments [7].

Given the multiple sources of plant germplasm, a key question concerns whether spontaneous urban vegetation is predominantly composed of native or exotic species, and which of these groups exhibits greater regenerative capacity following disturbance. More broadly, this raises the issue of the prevailing taxonomic composition of urban vegetation. Although some studies suggest that disturbance can limit the regeneration of alien invasive species [4], this topic remains debated and warrants further investigation.

The habitat analogue approach allows for the transfer and implementation of biodiversity from natural habitats into anthropogenic environments. When pedoclimatic conditions are comparable, germplasm from analogous habitats can be introduced with a high probability of successful establishment. Within this framework, several studies have documented cases in which rare plant species preferentially colonise urban settings, highlighting the potential role of cities in nature conservation, including the preservation of rare and threatened plant species [5].

Informal urban biodiversity develops independently of formal planning regulations, often driving unexpected transformations of urban spaces and generating socio-ecological value, as reflected in increasing recognition by local communities [8].

Spontaneous ruderal plants, in particular, may offer a foundation for low-cost, low-maintenance urban green infrastructure that is well-adapted to local climatic conditions. Beyond delivering a broad range of ecosystem services (ESs), these species are able to thrive in heavily human-modified environments and tolerate harsh conditions, including water scarcity and shallow or compacted soils [9].

The use of spontaneous urban vegetation for ornamental purposes and its valorisation to address the diverse needs of urban residents presents challenges in terms of care and management; nevertheless, it holds considerable potential as an innovative and sustainable solution [10]. Enhancing the aesthetic appeal of spontaneous vegetation may facilitate its adoption as an alternative or complement to conventional ornamental plantings in urban environments. From a design perspective, this approach entails the deliberate incorporation of spontaneous plant communities, potentially enriched through targeted plantings featuring showy flowers or fruits, diverse foliage textures, and distinctive growth forms. However, a careful assessment of competitive interactions is required to ensure the long-term coexistence and stability of these emerging plant assemblages [11].

We hypothesised that, although the existing literature on spontaneous urban plants largely adopts either a botanical approach (e.g., species inventories, native versus exotic composition) or an ecological perspective (e.g., functional traits, invasiveness), design-, cultivation-, and management-oriented studies aimed at their integration and valorisation within urban green infrastructure remain scarce. Beyond the recognition that numerous plant species spontaneously establish in cities and form communities of varying persistence and complexity, the potential to integrate this cost-free green heritage into urban green infrastructure—and to harness the ecosystem services (ESs) it provides—remains insufficiently explored.

On this basis, we formulated several research questions and conducted a comprehensive literature review on urban spontaneous plants with the aim of identifying both consolidated knowledge and existing gaps. Specifically, we sought to: (1) characterise the traits and functional types of urban spontaneous plants; (2) identify the main types of vegetation that develop spontaneously in cities; (3) determine which urban habitats are most frequently colonised; (4) assess the ecosystem services provided by spontaneous vegetation; and (5) explore design and management approaches capable of maximising the potential of urban spontaneous plant communities.

Urban wilderness represents a largely untapped, cost-free resource for urban populations, fostering closer interactions with nature, providing healthier urban spaces, and offering opportunities for environmental education. A deeper understanding of how spontaneous plants establish across different urban habitats, together with greater recognition of the ESs they deliver, can support increased awareness among planners and urban managers, promoting a shift towards more sustainable, nature-positive, and circular models of urban development.

## 2. Spontaneous Plant Traits, Functional Types and Vegetation

**Figure 1:**
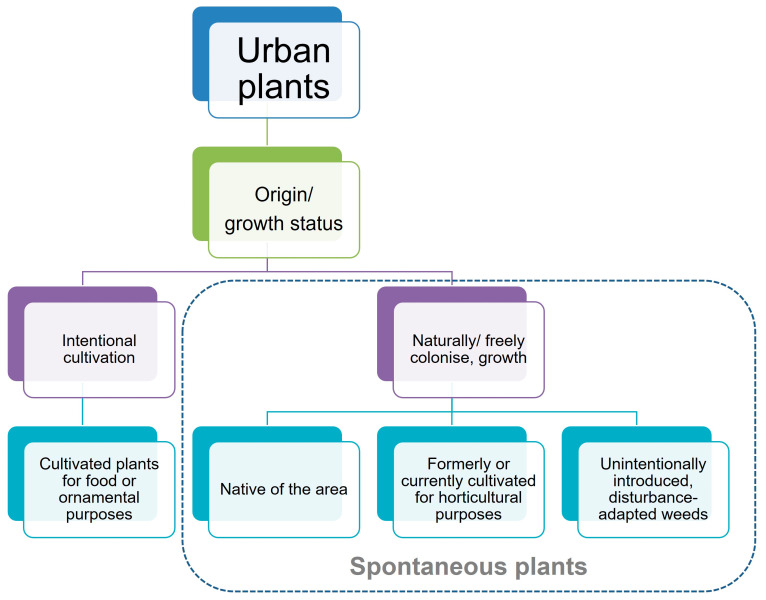
Urban plant classification, highlighting the group of spontaneous plants (adapted from [12]). In cities, both native and exotic plant species may arrive through intentional or unintentional introduction and, in some cases, encounter suitable conditions to establish and become naturalised. While cultivated plants require agronomical care, spontaneous plants are self-sustaining.

Urban spontaneous plants include all plant species that establish and grow autonomously—whether native or introduced intentionally or unintentionally by humans—and are not subject to cultivation [10,12] (Figure 1).

To understand the quantitative relationships between the characteristics of urban green spaces and the spontaneous plants accidentally introduced, Gao et al. [13] surveyed the city of Kunming, located in a biodiversity hotspot in southwestern China. Spontaneous plants were classified into native, non-native, and invasive. Of the 386 recorded species, 76.2% were herbaceous plants. Although invasive constituted a relatively small portion of the total species (17.9%), they accounted for six of the ten most common species.

Ruderal species are a key component of urban plant diversity and contribute to the sustainability of urban green spaces. Studies from Beijing show that native ruderals dominate across different land-use types, with higher species richness in less intensively managed areas. Ruderal diversity is influenced by factors such as distance from the urban centre, management intensity, and soil characteristics [14]. Common urban species are typically human-dispersed, nutrient-demanding, and adapted to dry soils; they do not follow the S-strategy, *sensu* Grime, and generally produce small seeds forming short-term persistent seed banks [15,16].

Spontaneous plants represent the most natural component of urban biodiversity, and the wilderness occurring in urban industrial areas constitutes an ideal ecosystem for investigating the mechanisms underlying the interaction between urbanisation and sites characterised by exceptionally high species richness [17]. Herbaceous plants, often co-occurring with small drought-adapted shrubs, act as pioneer colonisers of spaces exposed to anthropogenic disturbance, such as road verges, brownfields, and lawns. These species-rich ecosystems are widespread in urban areas but are frequently overlooked, despite their high biodiversity and the important ecological and social functions they can provide [18]. Urbanisation influences plant functional traits, although its effects are highly context-dependent. Along an urbanisation gradient from the periphery to the city centre, plant communities tend to become more specialised, with species characterised by short life cycles and high seed production that facilitate rapid population establishment. In peripheral areas, by contrast, assemblages are generally less specialised and dominated by stress-tolerant species [19]. Climatic and environmental stressors further shape plant adaptability: in Mediterranean regions, heat and water availability represent the main limiting factors, whereas in tropical urban areas soil pollution and habitat fragmentation play a dominant role. Several functional traits observed in spontaneous urban plants are associated with resilience to pollution, drought tolerance, and seasonal extremes. These adaptive features include sclerophyllous leaves, the presence of trichomes, deep rooting systems, phenological plasticity, dormancy mechanisms, high antioxidant activity, and root plasticity. Urban soils are often highly heterogeneous in terms of texture, structure, organic matter content, pollutant load, and seed bank composition, and this variability strongly influences the strategies of species capable of colonising and successfully establishing in these environments [18]. Urban herbaceous vegetation developing on infertile or disturbed soils is frequently characterised by a high proportion of legumes, including genera such as *Lotus*, *Trifolium*, *Medicago*, *Onobrychis*, *Galega*, and other *Fabaceae*. These pioneer species facilitate the establishment of additional plant species and functional types (e.g., grasses and forbs) through their ability to fix atmospheric nitrogen.

Another type of spontaneous herbaceous community commonly found in urban environments is the ruderal–nitrophilous assemblage, which develops on fertile soils. In relatively undisturbed conditions, this assemblage is typically dominated by large perennial or biennial species. By contrast, in highly disturbed environments—where soils are compacted by trampling or regularly tilled and mown—therophytes constitute the prevailing life form. Additionally, dispersal limitation plays a significant role in shaping vegetation composition in urban vacant lots [20].

Functional diversity within urban plant communities does not appear to be strongly associated with phylogenetic diversity. In a comparative analysis of plant communities from 32 major cities across ten countries, Lososová et al. [21] identified statistically significant but very weak positive relationships between phylogenetic diversity and overall functional diversity, as well as between phylogenetic diversity and the diversity in both species’ dispersal strategies and competitive abilities.

A review of 29 studies examining plant traits in urban floras indicates that some traits, such as woodiness, seed mass, and plant height, tend to increase with urbanisation, whereas others show variable or inconsistent responses and remain poorly studied. This variability likely reflects differences in the type and intensity of urban stressors and the combined effects of multiple stresses, which complicate the identification of distinct urban plant trait patterns [7].

Plant traits are strongly influenced by the habitat in which species establish. In wetlands of the Jiangyangfan Ecological Park in Hangzhou, China—designed according to a “wild state” concept—spontaneous plants were predominantly characterised by achenes and capsules, with zoochory playing a key role in shaping species composition and distribution [22]. Similarly, studies conducted in inner-city railway areas in Lublin (south-eastern Poland) and Lviv (western Ukraine) indicate that plant assemblages are predominantly composed of hemicryptophytes perennial species and C-strategists (including CR and CRS types). These communities are mainly insect-, self- or wind-pollinated, reproduce primarily by seed, and are dispersed by wind [23]. Railway areas provide favourable niches for spontaneous vegetation, characterised by dry, nutrient-poor soils and open spaces that experience limited human disturbance.

Surveys of spontaneous flora in urban parks indicate that species typical of ruderal habitats—namely disturbance-tolerant species, weeds, and ruderal competitors—account for the highest proportion of the flora. Native species are the most frequent component of spontaneous vegetation (exceeding 50%), although a relatively high ratio of archaeophytes to neophytes has also been reported [24]. Surveys conducted along the Hejiagou and Majiagou river corridors showed that the life-form spectrum of spontaneous vegetation is dominated by annual and biennial species. Native species accounted for 71.52% of the flora, while invasive alien species represented 18.54%. In terms of reproductive strategies, seed propagation was predominant, characterising 96.03% of the species. Dispersal occurred mainly through autochory (47.02%), followed by anemochory (34.44%), zoochory (29.80%), and hydrochory (13.91%) [25].

Reduced human disturbance in abandoned urban plots allows natural succession to develop, leading to spontaneous vegetation that supports urban ecological stability. Long-term surveys (2009–2022) in Xiyuhe (Beijing region) showed significant vegetation changes after 2018, with wind dispersal becoming the predominant seed dispersal strategy due to its efficiency over long distances. Although many species have small, inconspicuous flowers, their ornamental value derives mainly from the collective visual effect of plant communities rather than individual blooms [26].

The presence of natural vegetation in urban areas is strongly influenced by the model of urban expansion adopted. Compact urbanisation is generally regarded as a key strategy for sustainable urban development; however, an analysis of vegetation in degraded sites within the Buenos Aires metropolitan area (Argentina) showed that sprawl-type urbanisation had positive effects on local vegetation, as indicated by a higher recorded species richness [27].

Species richness in urban areas is influenced by site characteristics and human activities. In Izmir (western Turkey), roadside verges along the urban–rural gradient showed lower species richness and Shannon diversity than abandoned land and peri-urban grasslands. This pattern contradicts the expectation of lowest diversity at the urban end of the gradient and was attributed to the area’s relative resistance to disturbance, likely linked to the Mediterranean climate and long-term human influence [28].

Habitat loss and alterations to abiotic conditions significantly affect urban biodiversity. A study conducted in Debrecen (eastern Hungary) compared plant composition across three urban habitats—abandoned land, urban parks, and peri-urban grasslands—dominated by semi-natural grassland and ruderal species. Urban parks, representing the most intensively urbanised habitat, exhibited the lowest species richness and Shannon diversity. The proportion of weeds and disturbance-tolerant species peaked in the city centre, likely due to intensive trampling and soil disturbance, while central-city floras were characterised by greater drought tolerance, possibly associated with excess drainage. Nitrogen-fixing species were less frequent in urban parks and peri-urban grasslands than on abandoned land, reflecting recent soil disturbance. Non-native species were abundant in both abandoned land and peri-urban grasslands, despite differing disturbance regimes, whereas cosmopolitan species were most prevalent in urban parks. Continuous human disturbance and the dominance of non-native and cosmopolitan taxa placed native species at a competitive disadvantage, resulting in a lower proportion of natives in the city centre. Although these habitats contributed little to the conservation of rare or threatened species, they play an important role in preserving residual grassland vegetation and provide valuable opportunities for urban greening initiatives [29].

Correlations between soil physico-chemical and biological properties across different depths along a disturbance gradient were analysed in four types of spontaneous ruderal vegetation: open vegetation on disturbed soils, roadside vegetation, annual grasslands, and perennial herbaceous communities. The results showed that physico-chemical factors, particularly total organic carbon, bulk density, and nutrient-cycling performance, were the main drivers shaping vegetation composition [30].

The relatively low level of human disturbance in many abandoned urban plots allows, to some extent, natural succession and ecological processes to occur, resulting in landscapes dominated by spontaneous vegetation and contributing to the stability of urban ecological functions. To examine vegetation dynamics in such contexts, surveys were conducted over a 14-year period (2009–2022) in the village of Xiyuhe, in the Beijing region. Following the demolition of cement structures, tree species established rapidly, with wind dispersal emerging as the predominant mode of seed dissemination [26].

A study conducted in Berlin (Germany) examined the role of emerging urban forests—spontaneously developing forests characterised by the coexistence of native and non-native plant species—in enhancing urban biodiversity. Beyond providing habitats for a wide range of plant species, including some endangered taxa, these forests enable city dwellers to experience urban wilderness. The findings indicate that, under Northern European climatic conditions, emerging urban forests can successfully adapt to dynamic urban environments. Their integration into urban green infrastructure may therefore support sustainable urban development and complement traditional restoration and greening strategies [31].

Anthropogenic activities affecting urban natural matrices, including soil and vegetation, strongly influence plant community composition. Spontaneous plants can colonise most urban spaces where minimal resources such as soil, water, and nutrients are available. In densely urbanised centres, polluted and nutrient-rich soils typically favour nitrophilous annual herbaceous species, whereas the drier, nutrient-poor soils of urban peripheries are more often dominated by perennial plants. Along the urban gradient, spontaneous plant strategies shift accordingly: city centres are richer in ruderal, disturbance-tolerant annuals, while peripheral areas with derelict soils and vacant lots are characterised by stress-tolerant perennials. Overall, soil heterogeneity plays a key role in structuring urban vegetation by regulating the establishment of different spontaneous species and functional types (FTs).

## 3. Habitats: Walls, Wetlands, Brownfields, Drylands

**Figure 2:**
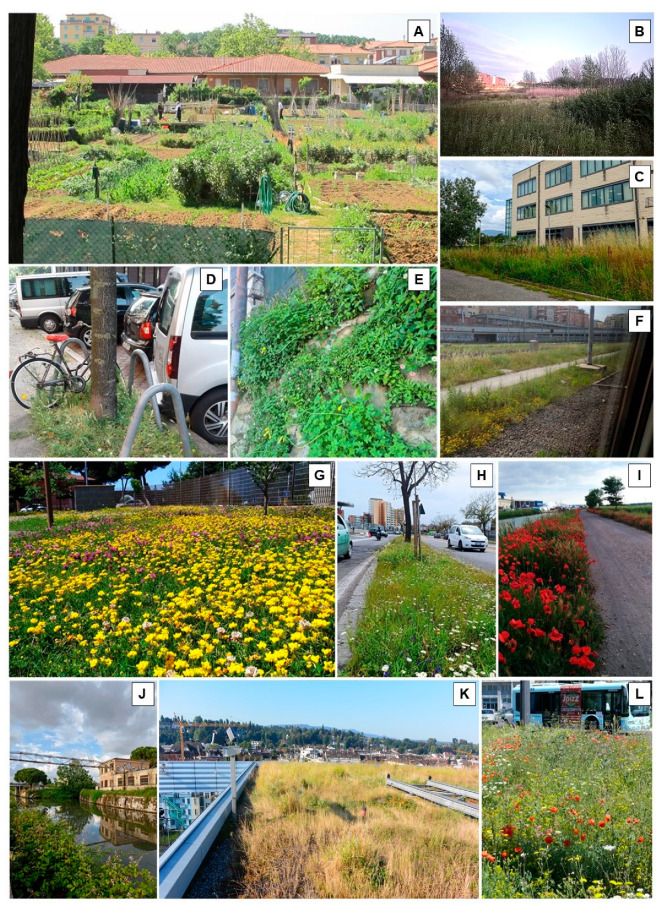
Urban habitats where spontaneous plants can spread and naturalise due to the low maintenance conditions. From top left: (**A**) community gardens; (**B**) wet areas; (**C**) industrial areas; (**D**) tree bases; (**E**) walls; (**F**) railways; (**G**) semi-natural lawns; (**H**) roadsides; (**I**,**J**) canal banks; (**K**) extensive green roofs; (**L**) roundabouts.

Spontaneous plants can establish across a wide range of urban habitats, and their occurrence is closely linked to patterns of land use (Figure 2; Table 1).

Urban green spaces provide important habitats for spontaneous plants, thereby enhancing biodiversity. Biodiversity conservation is strongly influenced by green spaces composition and gardening practices, and the coexistence of spontaneous vascular flora with ornamental plantings should be promoted. High biodiversity levels can be supported by both autogenous elements (such as groups of tall trees, copses, and wild grass communities) and semi-natural, intentionally sown elements like flowering meadows [32]. While landscape designers largely determine plant composition in urban parks, the diversity of artificially created habitat structures enables natural processes of spontaneous colonisation to occur [33].

Even small urban areas can support a high number of spontaneous plant species, thereby contributing to the enrichment and accumulation of urban biodiversity [48]. Although green spaces represent the primary source of plant propagules within cities, spontaneous plant diversity in urban environments is also strongly influenced by the presence and characteristics of buildings, which can either facilitate or constrain the dispersal and establishment of different species [34].

Herbaceous spontaneous vegetation can readily establish at the base of urban trees. A study conducted in Paris, France, showed that plant communities at tree bases vary in relation to multiple factors. Species abundance and distribution are influenced by biological traits such as seed longevity in the soil seed bank, as well as by tree-base characteristics, including trunk diameter, tree species, soil compaction, and the presence of animal droppings. Additional factors such as street orientation and neighbourhood structure— particularly proximity to green spaces—also play a role. Consequently, urban tree bases represent favourable habitats for certain species and make a meaningful contribution to the enhancement of urban biodiversity [35].

Urban allotment gardens deliver numerous ESs closely linked to floristic diversity. In a survey of 11 representative areas in Poznań, Poland (covering a total of 150 ha), 358 spontaneous plant species were recorded. These areas were characterised by high species richness and diversity, low levels of synanthropisation, and the presence of valuable geobotanical elements, including species of key importance under the European Community conservation guidelines. For these reasons, urban allotment gardens should be regarded as biodiversity hotspots for native species within the urban green infrastructure. Floristic composition is primarily influenced by the spatial and functional organisation of the gardens, as well as by their patterns of use [36]. A study conducted across 18 community gardens in Berlin, Germany, examined the diversity of both cultivated and spontaneous plant species. The results showed a positive correlation between the number of spontaneous species and cultivated species, while species richness was negatively correlated with the degree of urbanisation. These findings highlight that community gardens, beyond their role in urban food production, function as distinctive urban ecosystems in which cultivated and spontaneous flora coexist and jointly contribute to urban biodiversity [37].

Abandoned lots are often perceived as signs of urban decay, yet they can provide relatively undisturbed habitats for wildlife and deliver important ESs. Vegetation on brownfield sites emerges from the interaction of biophysical and social forces, and its study offers valuable insights into socio-environmental dynamics within cities. An analysis of vegetation patterns on brownfields in Chicago, Illinois (USA) by Anderson and Minor [38] revealed a high degree of species uniformity, indicating a limited presence of rare species. However, sites with greater amounts of waste showed higher species richness and uniformity, despite having smaller vegetated areas. Unmanaged areas represent valuable spaces for the conservation of urban biodiversity and promote interactions between citizens and nature by hosting spontaneous vegetation. However, the absence of regular management can lead to negative perceptions among residents [39].

By analysing wetlands that developed on former mining sites in the Silesian Uplands and the Kraków-Częstochowa Uplands, Błońska et al. [40] demonstrated that human-induced land transformation can lead to the creation of habitats that serve as refuge for organisms, particularly plant species of high conservation value within urban–industrial landscapes. Accordingly, the authors argue that anthropogenic wetlands should be integrated into urban planning frameworks and the management of industrial sites to enhance biodiversity conservation. Regarding environmental factors, a study conducted in a 20-hectare urban wetland ecological park in southern Hangzhou found that water depth and slope were the primary drivers shaping the diversity and spatial distribution of spontaneous plant species. In addition, the presence of cultivated plants significantly influenced the composition of spontaneous vegetation [22].

Rivers and canals are key components of urban ecology, supporting spontaneous vegetation and biodiversity. In Chengdu, China, species richness along river corridors varied with land use, urbanisation, and microhabitat conditions, with riverbanks and vacant watersides hosting the most diverse communities. These patterns highlight opportunities for nature-based solutions in urban rewilding and landscape design [41]. Urban river corridors in Harbin, China, host high spontaneous plant diversity, with vegetation composition varying across urbanisation gradients. Environmental and management factors—such as slope, proximity to water, vegetation structure, and maintenance intensity—strongly influence species life forms, distribution patterns, and dispersal strategies in the herbaceous layer [25]. In addition to green spaces, the ecological value of built-up areas should also be considered, as it is often overlooked despite these areas constituting the dominant form of urban land cover. Roads and pavements are ubiquitous linear structures present across all neighbourhood types and occupy extensive portions of urban landscapes. Bonthoux et al. [42] evaluated the capacity of pavements to support spontaneous flora through a floristic survey of 48 km of pavements in Blois, France. Over 300 species were recorded, with plant communities primarily shaped by pavement type, vegetation cover, and species richness, which was significantly higher on sandy than on asphalt pavements. Commercial and industrial areas exhibited greater species richness and cover than residential areas, while weeding frequency and nearby green spaces had limited influence. These findings highlight the importance of permeable pavements in supporting urban biodiversity, a factor that should be considered in urban planners.

Crevices in urban pavements can function as distinct microhabitats, characterised by significantly higher temperatures, pH values, electrical conductivity, and moisture content than adjacent roadside soils. These conditions create unique opportunities for the establishment of spontaneous plants. In Nashville, Tennessee (USA), 34 plant species were recorded growing in pavement cracks, of which only 12 were native, indicating a predominance of non-native species within these microhabitats [43].

Walls, the most widespread vertical surfaces in cities, can support diverse vegetation. Surveys in Chongqing, China, recorded 239 vascular plant species across 172 genera and 75 families growing on walls, with native species accounting for about 90% of the total. Wall characteristics were a key factor shaping the composition and structure of wall vegetation, exceeding the influence of surrounding environmental conditions. As such, walls represent valuable elements for enhancing urban biodiversity and supporting ESs in densely populated urban areas [44].

Road verges can provide important habitats for spontaneous flora. A study of 113 sites in the Czech Republic analysed renaturalisation processes across a successional gradient (1–42 years), linking species composition to site age, altitude, substrate texture, and surrounding forest cover. Herbaceous vegetation reached an average cover of 30% within two years, followed by shrub and tree establishment. Among the 320 recorded vascular plant species, there were 15 endangered species and 19 invasive aliens. Early successional stages were dominated by insect-pollinated species, highlighting the role of road verges as valuable resources for pollinators [45].

Blouin et al. [1] examined four anthropogenic microhabitats (wall bases, maintained and unmaintained fences, and hedges) along rural–urban gradients in Montréal and Québec (Canada). Species richness was unaffected by urbanisation in Montréal but declined in the most urbanised districts of Québec, while functional diversity increased with higher urbanisation levels. More urbanised contexts and disturbed microhabitats (wall bases and maintained fences) showed lower species richness and beta diversity. Overall, plant assemblage responses to urbanisation varied across species traits, cities, and microhabitats, underscoring the need to consider multiple spatial scales and habitat types when assessing flora homogenisation in urban environments.

In Hong Kong, a tropical wetland green roof was monitored for two years to assess spontaneous colonisation of plants and birds. Ninety-four spontaneous vascular plant species were recorded, mainly dispersed by birds and wind, with a minor contribution from the soil seed bank. Plant composition changed dynamically over time and included dominant ruderal species, woody plants, and hygrophilous herbs. As vegetation cover increased, species diversity and evenness also changed, and most vegetation parameters were positively correlated with bird community indices. These results demonstrate that extensive green roofs can support both plant and bird communities and play a meaningful role in urban ecology and conservation, even in densely built environments [46].

Although green roofs generally support fewer species than ground-level habitats and cannot replace them, their structural diversity—such as variation in substrate depth—can enhance species richness and abundance when suitable conditions are provided [47]. In densely urbanised areas, small, vegetated spaces such as roadside verges, derelict plots, and walls can harbour high levels of biodiversity [48]. Despite their limited size, these informal urban green spaces can deliver essential ESs to urban residents [49].

Various types of urban spaces and land use that support spontaneous vegetation have attracted increasing attention. However, a unified classification of these green areas is still lacking, as multiple terms are often used to describe essentially similar spaces and uses. Establishing a standardised framework would improve clarity and facilitate a better understanding of the relationships between spontaneous plants and urban green areas. Harsh habitats, such as pavement cracks, green roofs, walls, are subject to be colonised by highly invasive anemophilous species.

## 4. Ecosystem Services Provided by Urban Spontaneous Plants and Vegetation

Plants in urban areas provide a wide range of ESs, including improving environmental quality, mitigating the negative effects of human activities, and fostering a sense of place and cultural identity [50]. While these benefits are recognised for ornamental plantings, the positive contributions of spontaneously growing plants are often overlooked, with attention frequently focused on their perceived damage to built structures and historical monuments. Urban grasslands, too, play a crucial role in supporting biodiversity and maintaining ecosystem functions, making them essential for human well-being [28]. The coexistence of plants and humans in urban environments is considered feasible only when appropriate management measures are implemented to prevent damage to built structures [51]. To synthesise the main attributes of urban spontaneous plants, we conducted a SWOT analysis (Table 2) based on both the scientific literature and personal expertise.

The expansion of poorly managed and derelict land, often driven by population decline and infrastructure removal, poses a recurring challenge in many shrinking cities. Low management intensity creates conditions that favour plant colonisation, establishment, and succession, giving rise to distinctive urban habitats. Spontaneous urban vegetation can deliver multiple ecological and social benefits, but it may also generate disservices, including damage to buildings, the presence of unwanted fauna (e.g., disease vectors), and negative perceptions associated with its unmanaged appearance [53]. Notably, attitudes towards spontaneous urban vegetation appear to be strongly influenced by cultural and geographical context.

Spontaneous vegetation plays a crucial role in conserving urban biodiversity and sustaining urban ecosystems [55]. Nature-based Solutions (NbSs) are increasingly recognised as an approach that harnesses natural processes to enhance ES provision and improve urban quality of life. Among the most widely implemented NbSs is rewilding, defined as the intentional reduction or abandonment of management in cultivated green spaces to promote wild nature and native plant communities [72].

Brownfields provide a range of ESs that address key urban challenges, including biodiversity conservation, climate change adaptation, and the promotion of healthy, recreational environments. However, these benefits can only be fully realised if brownfield sites are recognised and integrated as essential components of urban green infrastructure [62]. Spontaneous plants can contribute to the reduction in atmospheric particulate matter (PM). A study conducted in Tarnów, Poland, showed that roadside vegetation combining herbaceous plants, shrubs and trees was most effective in PM removal [73]. In addition, spontaneous herbaceous species have demonstrated the capacity to bioaccumulate polyfluoroalkyl substances (PFASs), highlighting their potential role in mitigating urban pollution [57].

When maintenance of green roofs is reduced due to cost constraints, spontaneous plants may colonise these systems and, in some cases, coexist with and complement the original plantings. When sufficient vegetation cover is established, green roofs can deliver key ESs such as stormwater mitigation, habitat provision, and climate regulation. Spontaneous species are often able to establish without irrigation or fertilisation, thereby reducing management costs. In hot and dry climates, many species can successfully colonise shallow substrates and regenerate from seed, making this approach particularly suitable and cost-effective [67]. To assess the role of spontaneous vegetation in stormwater mitigation on green roofs, a 100-day rainfall simulation with alternating wet and dry cycles was conducted in Melbourne, Australia, using 14 plant species that naturally colonise green roofs in Mediterranean-type climates. The results indicated that spontaneous vegetation enhances rainwater retention compared to bare substrates and performs similarly to commonly used green-roof species. However, the contribution of spontaneous plants to stormwater mitigation depends largely on other key factors, particularly substrate depth and rainfall characteristics [74].

**Figure 3:**
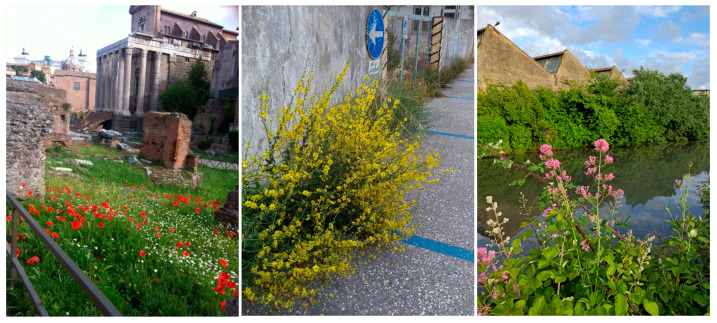
Archaeological monuments, wall bases, and canals create habitats where spontaneous plants can establish and flower due to low disturbance and elevated temperatures. In these settings, spontaneous vegetation enhances the landscape with its ephemeral aesthetic, creating a striking contrast between natural elements and built heritage.

A study of natural vegetation in urban green spaces in a Mediterranean city examined plant biodiversity, soil carbon sequestration, nutrient cycling, and water regulation across four vegetation types: trampled-soil vegetation, roadside vegetation, annual grasslands, and perennial herbs. Perennial herbs showed the highest biomass and were associated with soils rich in organic carbon and available phosphorus, while annual grasslands exhibited the greatest plant diversity. Roadside vegetation was characterised by higher phenoloxidase activity than trampled-soil vegetation and annual grasslands. Overall, plant community composition emerged as a strong indicator of ecosystem functions and services, which were unevenly distributed across urban habitats [75]. Urban wilderness offers aesthetic value through colour and form, as well as a sense of naturalness, which has been identified as a particularly strong driver of human preference [52] (Figure 3).

In a survey of spontaneous vegetation in Beijing Olympic Forest Park, 21 distinct vegetation types were identified and their aesthetic roles evaluated. The results showed that most spontaneous species were harmoniously integrated with cultivated plantings and that urban parks contain numerous microhabitats that are readily colonised by spontaneous plants [58].

Residents’ perceptions of urban biodiversity are strongly influenced by sociocultural factors. A survey of 1015 residents in Beijing, China, showed that appreciation of spontaneous vegetation varied with background, with professionals, individuals with higher education, and those having greater exposure to nature more likely to recognise its aesthetic and ecological value and to support its conservation in urban green spaces [61]. These findings underscore the importance of environmental education in improving public attitudes, facilitating biodiversity-friendly design, reducing management costs, and increasing access to more natural urban environments. At the same time, spontaneous vegetation can evoke positive sensory and experiential responses (“landsenses”) across educational levels [54]. Public perceptions of brownfield sites with natural vegetation also vary, but greater acceptance of their use as green spaces could enhance biodiversity, promote direct experiences of nature, and improve residents’ satisfaction and overall urban quality of life [62].

Research quantifying the ESs provided by spontaneous plants in urban environments remains limited, as studies have largely focused on cultivated ornamental species—particularly trees—owing to their prominent role in urban greenery. However, the available, albeit fragmented, evidence suggests that spontaneous vegetation plays a meaningful role and represents a research area deserving targeted and systematic investigation.

## 5. Possible Management Strategies in Relation to Land Use

In some cases, wastelands have been successfully repurposed for sports, recreation, education, and leisure, as exemplified by Landschaftspark Duisburg-Nord (Ruhr, Germany), a former industrial site converted into a public park. In more urban contexts, however, wastelands have generated fear and a sense of insecurity, leading to their clearance and redevelopment for more economically profitable uses. More recently, these “unintentional landscapes” have increasingly been incorporated into urban design—or deliberately emulated through aesthetic approaches inspired by wastelands—highlighting the need for appropriate and context-specific management strategies [76].

A survey of 190 urban land-use types in Haikou, China, found that cultivated plant richness was highest in colleges and universities, research areas, and high-density residential zones, whereas spontaneous plant richness was greatest in hospitals, colleges and universities, and low-density residential areas. Maintenance frequency (e.g., weeding and pruning) was positively correlated with spontaneous plant richness, and proximity to freshwater sources further increased the number of spontaneous species [77].

Intensive horticultural practices have contributed to excessive resource use and reduced ecological functionality in urban green spaces, prompting growing interest in spontaneous vegetation. Effective management of spontaneous plants requires a better understanding of their biodiversity, functional roles, and spatial distribution within urban parks, with the aim of developing sustainable, low-maintenance green space designs [58]. Importantly, unmanaged spontaneous vegetation can equal or even surpass planted green infrastructure in fulfilling key ecological functions within cities [10].

Urban ecosystems, initially shaped by landscape planning, design, construction, and management, continue to evolve under these influences. This ongoing dynamic process generates long-term ecological effects and promotes complex biodiversity patterns characterised by the coexistence of cultivated and spontaneous species [12]. Urban ecosystems, initially shaped by landscape planning, design, construction, and management, continue to evolve under these influences, generating long-term ecological effects and complex biodiversity patterns characterised by the coexistence of cultivated and spontaneous species. In a study of 38 urban parks in the high-density city of Shanghai, China, Chen et al. [12] recorded 211 spontaneous plant species across 169 genera and 82 families. Spontaneous plant diversity increased with park area, while site density influenced species composition. At the site scale, water conditions, vertical vegetation stratification, low altitude, tree cover, and the presence of cultivated plants significantly shaped the diversity and composition of spontaneous plant assemblages.

Spontaneous urban vegetation is often undervalued in city planning. A study of 13 representative Italian cities showed that spontaneous woodlands—varying in size, age, and former land use, mainly residential and industrial—were excluded from more than half of official urban plans. Instead, planning documents frequently proposed alternative land uses involving new soil sealing and tree planting, rather than conserving existing woodland patches. Only four case studies partially recognised spontaneous woodlands as components of urban green infrastructure [66].

Public awareness of the benefits provided by spontaneous urban plants remains limited. An online survey of 708 respondents in Latin America showed a general preference for formally designed green spaces over areas dominated by native vegetation. However, when participants were informed about the ecological benefits of native plants, their preferences temporarily shifted in favour of more naturalistic spaces. Once this information was no longer salient, preferences reverted to formal green spaces, underscoring the short-term effect of awareness and the need for sustained environmental education [78].

Spontaneous plant communities vary with maintenance regimes in terms of species composition. In 13 urban parks in Beijing’s Xicheng District, traditional parks were dominated by annual and biennial species, whereas semi-natural parks are characterised by perennial communities. Significant differences in species homogeneity were also observed, with large traditional parks showing the highest average homogeneity [79].

Unused urban land is increasingly recognised for its potential to support biodiversity and enhance residents’ quality of life, though these benefits are strongly shaped by management practices and urbanisation-driven soil degradation. Soil quality, often overlooked in sustainable urban planning, plays a key role in sustaining urban biodiversity and ecosystem functioning. A study in Cleveland showed that vegetation success in vacant lots depended on soil biota and management intensity: monthly mowing of spontaneous herbaceous vegetation at 15–20 cm resulted in higher biomass and more abundant wildflower blooms than annual autumn mowing. These findings highlight the need to integrate above- and below-ground habitat management to improve the ecological performance of urban green spaces [80]. Urban green space management should promote natural ecological processes to foster biodiversity, support naturalisation, and create synergies between urban design and management objectives [81]. In Mediterranean contexts, management practices play a crucial role in spontaneous herbaceous vegetation by limiting soil compaction while enhancing biodiversity, carbon sequestration, and water regulation [75].

A study of spontaneous vegetation in residential green spaces in Fuzhou, China, found a distinct flora compared with other urban environments, characterised by a high proportion of non-native species (43.77%), largely driven by residents’ activities and the cultivation of ornamental and edible plants. Wild plant composition and distribution were influenced by maintenance intensity, the urbanisation gradient, and the proportion of vegetated soil within green spaces. In addition, residential socio-economic factors—including building age, housing prices, and population density—significantly affected spontaneous plant assemblages [82]. An analysis of spontaneous plants diversity in Beijing’s urban parks showed that maintenance intensity, perimeter-to-area ratio, and green-space fragmentation were all negatively correlated with spontaneous plant abundance [68].

Soil seed banks contribute to the temporal continuity and successional dynamics of vegetation in abandoned urban lots, making their composition and dynamics key to understanding spontaneous plant establishment. Species frequency in the upper soil layer were positively correlated with those in deeper layers and with above-ground vegetation. Moreover, small lots in densely populated neighbourhoods contained higher densities of weed seeds in the topsoil than larger lots in less densely populated areas [59].

An analysis of urban green spaces in Debrecen (eastern Hungary) and their connectivity with the surrounding Regional Ecological Network showed that 65% of functional green spaces are potentially connected, facilitating the dispersal of species typical of semi-natural open habitats [24]. Accordingly, natural succession could be more widely incorporated into the restoration of roadside verges, as an alternative to tree planting or the use of species-poor seed mixtures [45].

Efforts to enhance the aesthetic appeal of abandoned urban areas can inadvertently lead to substantial biodiversity loss. This was demonstrated in Rome (Italy), where spontaneous revegetation of a disused landfill supported an exceptionally high vascular plant richness (269 species within 0.2 km^2^), dominated by Mediterranean annuals and closely reflecting the regional species pool. Although the site was designated an Urban Nature Reserve following public advocacy, subsequent conversion to conventional neighbourhood park management led to a 50% decline in species richness and an 80% reduction in the steno-Mediterranean component, resulting in biotic homogenisation. The removal of demolition debris was likely a key driver of these changes. Similarly, summer irrigation used to prevent vegetation stress can further promote floristic homogenisation by favouring a limited set of species [71].

In the built-up area of Guiyang, southwestern China, spontaneous plants were analysed to assess patterns of diversity, distribution, and responses to urban environmental factors. Species richness and the Shannon–Wiener diversity index declined with increasing urbanisation, although no clear urban–rural gradient in species distribution was observed. Plant diversity was significantly influenced by building age, slope, and land-use type, while species richness was primarily associated with elevation and demographic change [83].

Promoting spontaneous vegetation in highly urbanised environments may face resistance from planners, managers, and urban users. An integrated approach is therefore required to address the interconnections between ecological, psychosocial, governance, and management dimensions. Providing on-site ecological information (e.g., signage) can moderately improve public perceptions of streetside spontaneous vegetation, but this educational strategy should be complemented by additional measures to reinforce and sustain positive attitude change [63].

Spontaneous urban plants are particularly effective in mitigating environmental challenges due to their capacity to sustain urban biodiversity and deliver ecological benefits with minimal maintenance inputs. Within an adaptive governance framework, observing spontaneous vegetation can inform planting decisions, support the monitoring of vegetation dynamics, and help conserve existing plant communities. Integrating spontaneous plants with targeted plantings may further enhance these benefits while addressing long-term maintenance constraints [84].

To enhance aesthetic appreciation of spontaneous plants, a project led by the New York–based Future Green Studio engaged the public through social media, both to raise awareness and to crowdsource data on geolocation and ecological characteristics. This approach resulted in the development of an open, interactive digital database covering New York City and achieved higher levels of stakeholder engagement than traditional outreach initiatives [64].

Biodiversity richness is closely linked to maintenance intensity. A systematic review of 92 studies revealed a complex relationship between maintenance practices and biodiversity, largely depending on the type of intervention and the metrics used to assess biodiversity. Mowing was the most frequently examined practice, with higher mowing intensity generally showing a negative association with plant abundance and species richness [69,70]. In the context of green roofs, a study in Avignon (southeastern France) showed that planting perennial species (e.g., *Sedum* L. spp., *Iris lutescens* Lam.), followed by spontaneous colonisation, is more effective for the long-term persistence of extensive, non-irrigated green roofs in Mediterranean climates than sowing species-rich mixtures dominated by annual Mediterranean plants [85].

**Figure 4:**
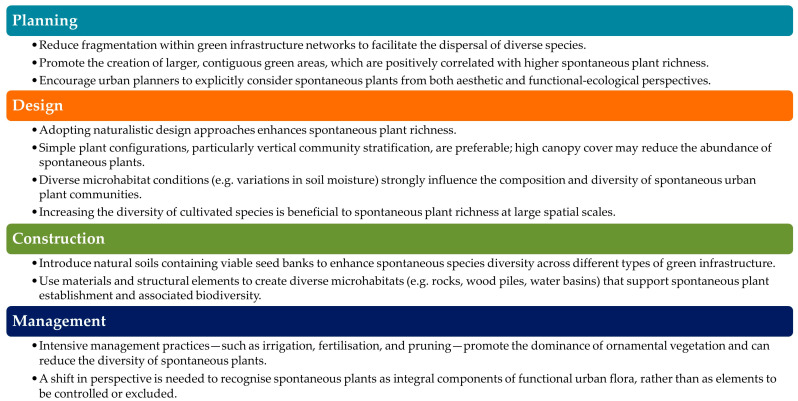
Strategies to enhance the presence of spontaneous plants in urban green spaces derived from the findings of the review.

Floral diversity and composition in urban parks are shaped by a combination of socio-economic, environmental, and ecological factors, resulting in distinctive urban habitats composed of both spontaneous and planted species [86]. To manage this “new urban wilderness” effectively, it is essential to move beyond the native–exotic dichotomy and focus instead on ecosystem functionality and ecological performance [10]. Moreover, these plants can be cultivated sustainably in private gardens and natural areas across both urban and rural contexts, owing to their high ecological adaptability and resilience [52]. To promote the integration of spontaneous plants in urban environments, actions are required at multiple stages, from spatial planning to the management of green spaces (Figure 4).

## 6. Materials and Methods

**Figure 5:**
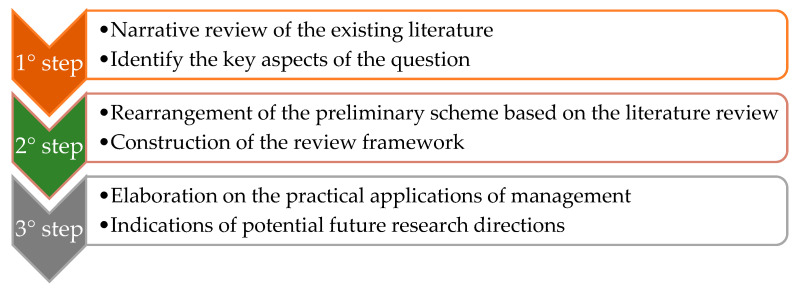
Main steps of the review process.

The aim of this literature review was to examine the characteristics and contribution of plants and spontaneous vegetation in urban areas, the ecosystem services they provide, and the extent to which management strategies can enhance their ecological functioning. To this end, a narrative review of the existing literature was first conducted to identify and synthesise the key issues addressed (Figure 5).

To ensure broad coverage, the literature review was conducted using Web of Science, Scopus, and Google Scholar, covering the period from 2015 to March 2026. Search terms included “spontaneous plants and/or vegetation” and “urban area/city”, “spontaneous plant diversity and urban contexts”, “composition profile of urban spontaneous plants”, “urban plant biodiversity”, and “composition differences in urban spontaneous plants”. All terms were combined using the Boolean operator “AND” with “ornamental plant” to ensure relevance to the study focus.

**Figure 6:**
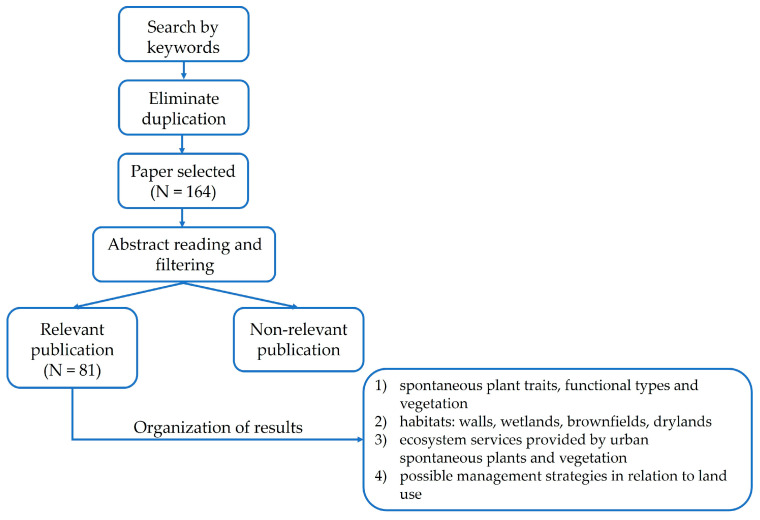
Literature review process.

Inclusion criteria comprised publications written in English (or with an English abstract) and included peer-reviewed articles, books, and policy documents. The literature review was structured around specific thematic topics, as illustrated in Figure 6.

The findings were used to clarify the role of spontaneous plants in urban environment and to formulate management recommendations aimed at enhancing their value, increasing public knowledge, and improving residents’ acceptance of spontaneous vegetation.

## 7. Conclusions

Urban areas host spontaneous plants interwoven with cultivated vegetation, contributing to urban ecosystem functioning and offering benefits to residents. Within these novel habitats, native and non-native species coexist and provide ecosystem services comparable to those of planted green infrastructure, with the added advantage of a minimal environmental footprint, as they are neither planted nor intensively managed. Sensitive and informed management can conserve and enhance spontaneous urban vegetation, delivering multiple benefits, foremost among them the development of stable and resilient plant communities.

In addressing the aims of this review, spontaneous plants were found to be shaped— in terms of traits and functional types—by urban stressors such as poor soil quality, pollution, drought, and disturbance. City centres are typically dominated by short-lived species with rapid life cycles, whereas peri-urban vacant areas tend to support perennial and woody vegetation. Vegetation type is largely determined by habitat characteristics and disturbance intensity, whether accidental or intentional, often resulting in early successional stages. Although spontaneous plants can colonise all urban spaces, their presence is enhanced by open areas, low management intensity, and proximity to cultivated vegetation. Extreme environments (e.g., walls, green roofs, and infertile soils) may also host highly specialised species. Spontaneous plants provide ecosystem services comparable to those of cultivated vegetation, with the added advantage of reduced planting and maintenance costs, although potential disservices include damage to built structures and perceptions of neglect. From a design and management perspective, a key limitation remains the limited knowledge base and cultural recognition of spontaneous plants, which are rarely described or integrated into conventional horticultural frameworks.

Future research should focus on disentangling the ecological and socio-environmental drivers shaping spontaneous plant communities in cities, with particular attention to intraspecific trait variability, successional dynamics, and habitat specificity along urbanisation gradients. Comparative studies across biogeographical regions, combined with integrated analyses of environmental conditions, management practices, and soil biota, are needed to better understand community assembly processes. In parallel, the role of private gardens and residents’ practices in facilitating spontaneous plant dispersal remains underexplored. Finally, advancing knowledge on social perceptions of spontaneous vegetation across cultural contexts is essential to support its broader acceptance and integration into urban design, planning, and biodiversity-oriented management strategies.

Enhancing the presence of spontaneous plants in urban environments requires moving beyond the conventional “first cleaning, then greening” paradigm [87] and adopting a multidisciplinary approach. Owing to their minimal horticultural requirements, spontaneous vegetation represents a sustainable strategy for increasing urban plant cover. Public acceptance can be improved through targeted, low-intensity interventions that enhance aesthetic quality without compromising ecological value. Nevertheless, spontaneous vegetation must be managed in ways that respect its ecological characteristics while preventing the spread of invasive species. This shift calls for moving beyond an overly anthropocentric perspective towards a holistic understanding of the benefits of spontaneous vegetation. Accordingly, further research is needed to identify management practices that effectively balance ecological integrity, risk control, and social acceptance. Environmental education and clear, consistent communication strategies are also essential to improve public perception and promote the integration of spontaneous vegetation within urban green space management.

## Figures and Tables

**Table 1:** Different urban habitat/land use typologies related to the traits of spontaneous plants and/or vegetation.

Habitat Typologies	Kind of Spontaneous Plants and/or Vegetation	References
Urban parks	Groups of tall trees, native shrubs and epiphytes, wild grass, flowering meadows	[32,33,34]
Tree bases	Plants able to assure a soil bank and adapt to stress (soil compaction) and disturbance (trampling)	[35]
Allotments/ community gardens	High plant diversity; hemicryptophytes, presence of native and rare plant species	[36,37]
Vacant lots Brownfields	High variability of plant species diversity and functional types; late successional stages; vegetation not maintained may induce negative perception	[38,39]
Wetlands	Plant species crucial for conservation (peat bog vegetation); calciphilous species; native plants with peculiar characteristics of the fruit that facilitate zoochory diffusion	[22,40]
River corridors	Great variability of species; rare occasional species, annuals and biennials, high percentage of native plants, autochory	[25,41]
Road and pavement cracks	Plants influenced by the surrounding vegetation; higher diversity in permeable pavements, ruderals, non-native, invasive	[42,43]
Walls	Plants determined by the wall characteristics and the surrounding vegetation; high presence of herbaceous plants	[44]
Road verges	High percentage of insect-pollinated species in early successional stages; woody plants in late successional stages	[45]
Green roof	Anemochory and zoochory; herbaceous and ruderal plants in early stages, woody plants in late stages depending on the soil dept	[46,47]
Railways	Anemochory, stress tolerators, herbaceous plants	[23]
Hedgerows	Shade tolerant, Biotic pollination	[1]
Wall bases	Therophytes, late flowering	[1]

**Table 2:** SWOT (strengths, weaknesses, opportunities, threats) analysis of the spontaneous plants in urban areas.

**STRENGTHS**	**WEAKNESSES**
✓ Spontaneous plants are the most natural component of urban biodiversity [17] and can act as urban ecological indicators [30]. ✓ Spontaneous plants are “already there”, adapted to the conditions and have all the advantages of growing from seed (ecological plasticity, genetical variability, good root apparatus with taproot) [52]. ✓ Urban spontaneous vegetation provides a range of ecological, cultural and sociological benefits via ESs, such as supporting urban wildlife, enhancing regulatory ecosystem functions and services, reducing pollution, connecting people with nature, and improving human health and well-being [38,48,52,53,54,55,56,57,58]. ✓ Spontaneous urban vegetation creates unique landscapes in cities and can evoke positive senses, such as naturalism and nostalgia (landsenses) associated with the varied colours of flowers and fruits [53,54]. ✓ Urban spontaneous vegetation (urban wilderness) is costless and reduces the carbon footprint [6]. ✓ Soil seed bank guarantees the temporal continuity and succession of vegetation on abandoned urban lots [59].	✓ Spontaneous plant diversity in urban areas depends on the presence and characteristics of buildings, which influence the ability of different species to spread [34]. ✓ Spontaneous plants are highly sensitive to impermeable paving, which reduces their presence and thus urban biodiversity [42]. ✓ Spontaneous urban vegetation can also harbour unwanted animals, such as disease vectors [53]. ✓ In some cases, due to untidy appearance, spontaneous vegetation may evoke negative emotions in people [53,60]. ✓ Appreciation of the aesthetic characteristics of spontaneous plants is related to the sociocultural background of the inhabitants [61,62,63,64]. ✓ The lack of knowledge regarding biodiversity and the distribution patterns of spontaneous vegetation in urban parks reduces its use in green spaces [65]. ✓ There is a lack of emphasis in urban planning on plant communities that spontaneously establish themselves in cities [66].
**OPPORTUNITIES**	**THREATS**
✓ When exposed to relatively low levels of human interference, native plants can facilitate natural succession and ecological processes, thereby enhancing the stability of urban ecological functions [26]. ✓ The integration of emerging urban forests into urban green infrastructure can help create sustainable cities [31]. ✓ Autogenous (spontaneous) elements (groups of tall trees, small woodlands, communities of tall grasses) can be combined with semi-natural anthropogenic elements (flowering meadows), which could ensure a relatively high level of biodiversity [32]. ✓ The ability of spontaneous native species to establish on green roofs without the need for irrigation or fertilisers reduces maintenance costs, thereby encouraging the adoption of this green infrastructure [67].	✓ Although invasive species make up a relatively small proportion of the total number of spontaneous plant species, they are often the most common [13]. ✓ The high percentage of alien and cosmopolitan species, combined with ongoing human disturbance, puts native species at a competitive disadvantage [29]. ✓ In the absence of appropriate management practices, damage to buildings may occur [51]. ✓ Spontaneous plants are affected by excessive maintenance, low perimeter-to-area ratios, and the fragmentation of green spaces [68,69,70]. ✓ The desire to enhance the aesthetic appearance of an abandoned area can lead to a significant reduction in biodiversity; certain cultivation practices, such as irrigation, result in the homogenisation of vegetation [71].

## Data Availability

No new data were created or analyzed in this study.
